# Lightweight Soft Robotic Glove with Whole-Hand Finger Motion Tracking for Hand Rehabilitation in Virtual Reality

**DOI:** 10.3390/biomimetics8050425

**Published:** 2023-09-14

**Authors:** Fengguan Li, Jiahong Chen, Zhitao Zhou, Jiefeng Xie, Zishu Gao, Yuxiang Xiao, Pei Dai, Chanchan Xu, Xiaojie Wang, Yitong Zhou

**Affiliations:** 1 Shien-Ming Wu School of Intelligent Engineering, South China University of Technology, Guangzhou 510641, China; 202030010084@mail.scut.edu.cn (F.L.); 202030010015@mail.scut.edu.cn (J.C.); 202030010275@mail.scut.edu.cn (Z.Z.); 202030010190@mail.scut.edu.cn (J.X.); 202030010039@mail.scut.edu.cn (Z.G.); 202030010183@mail.scut.edu.cn (Y.X.); 202120160303@mail.scut.edu.cn (P.D.); 2 The Institute of Intelligent Machines, Hefei Institutes of Physical Science, Chinese Academy of Sciences, Changzhou 213164, China; xuchan@mail.ustc.edu.cn (C.X.); xjwang@iamt.ac.cn (X.W.)

**Keywords:** soft robotic glove, hand rehabilitation, twisted string actuator, finger motion tracking, virtual reality

## Abstract

Soft robotic gloves have attracted significant interest in hand rehabilitation in the past decade. However, current solutions are still heavy and lack finger-state monitoring and versatile treatment options. To address this, we present a lightweight soft robotic glove actuated by twisted string actuators (TSA) that provides whole-hand finger motion tracking. We have developed a virtual reality environment for hand rehabilitation training, allowing users to interact with various virtual objects. Fifteen small inertial measurement units are placed on the glove to predict finger joint angles and track whole-hand finger motion. We performed TSA experiments to identify design and control rules, by understanding how their response varies with input load and voltages. Grasping experiments were conducted to determine the grasping force and range of motion. Finally, we showcase an application of the rehabilitation glove in a Unity-based VR interface, which can actuate the operator’s fingers to grasp different virtual objects.

## 1. Introduction

Active rehabilitation training can benefit patients with hand injuries, neurological or musculoskeletal conditions, and those who have undergone hand surgery by improving muscle strength and overall physical condition. However, traditional training with a physiotherapist is expensive, and most physiotherapy mechanical devices only allow patients to perform simple movement training, which is often perceived as monotonous and boring, leading to reduced patient motivation. Moreover, these rehabilitation devices are mostly bulky and heavy, so are not suitable for home rehabilitation. Therefore, there is a need for a low-cost, portable, and entertaining rehabilitation glove.

One widely used technology in rehabilitation equipment is that of exoskeletons. These can be categorized into hard exoskeletons, which use rigid joints, links, and motors to drive finger movements, and flexible exoskeletons, which are designed around a glove for improved wearability. Hard exoskeletons [1,2] provide high force output but their size and weight make them inconvenient and pose a risk of secondary injury. Some research has been undertaken on hand/wrist exoskeletons [3], but they are still too bulky and heavy.

To address the limitations of hard exoskeletons, soft exoskeletons designed as gloves with peripheral actuation are a suitable solution. These exoskeletons are lightweight and comfortable to wear. Soft exoskeletons can be categorized based on actuation sources, among which pneumatic and motor-tendon-based actuation methods are the two most popular types. Current pneumatic-driven hand exoskeletons [4,5,6] normally have a pneumatic chamber made of silicone or other flexible material, which is fixed to the back of the fingers of the glove and bends upon inflation of the pneumatic pump, thus bending the fingers. Although it is simple to control, this method has the drawback of bulkiness due to the air pump drive source and pneumatic chamber. In addition, the bulky actuators on the fingers may cause interference between fingers upon actuation. In contrast, the motor-tendon driven method is much lighter, where a small motor can be connected to the fingertip via a tendon and the finger can be driven by the rotation of the motor [7,8,9]. This method has a much simpler glove design, which makes the hand-wearable part more comfortable and lighter. Twisted string actuators (TSA) [10,11] are a type of motor-tendon actuator that converts a motor’s rotational motion into linear motion using a twisted string or wire. Compared to regular motor-tendon actuators, this method can provide five times the actuation force of spool-based motor-tendon actuators, which allows for using smaller motors as actuation sources [12], resulting in a much smaller robotic glove system. Tsabedze et al. [12,13] developed soft robotic gloves for rehabilitation purposes that are small in size and lightweight. Hosseini et al. [14] developed a force-feedback haptic glove using two independent TSAs and force sensors. However, these TSA gloves are mainly initial demonstration versions lacking posture-sensing functionality. Robotic gloves without posture-sensing capability cannot sense the finger state or record the rehabilitation process, making it difficult for robotic control and for doctors to assess a patient’s recovery progress.

Data gloves with hand movement tracking are effective for assessing hand training and developing control strategies for rehabilitation. Various sensors have been investigated for use in data gloves, such as flexible bending sensors, inertial measurement units (IMUs), etc. Flexible bending sensors are highly resistive sensors that have the advantage of being lightweight, small and inexpensive. However, such sensors can be easily damaged and their costs can be high [15]. IMUs are accurate and inexpensive and can collect information on each joint by placing a corresponding sensor on each finger segment. A number of IMU-based data gloves have been developed [16,17,18,19] and have shown high accuracy for hand motion tracking.

The monotonous and repetitive nature of hand rehabilitation training can be demotivating for patients undergoing physical therapy. However, the introduction of hand VR rehabilitation systems has revolutionized the process by adding an element of fun to the training. Additionally, these systems allow patients to train at home, making it more convenient for them [20,21]. Pereira et al. [22] developed a VR game called StableHand, which features farm-based scenarios that provide contextualized elements to support rehabilitation tasks. The game received a high system usability score (SUS) of 84.3 and positive feedback from seven participants, indicating its potential to enhance hand rehabilitation therapy.

In this study, we propose a VR rehabilitation training glove system. This glove can drive the users to grasp various virtual objects while the users can visualize their grasping in a virtual environment. This glove has two main functions: actuation-assisted rehabilitation and real-time gesture recognition. To assist with grip training, a soft robotic glove was developed using five TSAs. Each TSA is composed of a motor and a pair of strings on the arm. The TSAs apply tension to the fingertips, yielding finger bending and helping the patient with their grip exercises. Elastic bands are attached to the back of the glove to help restore the patient’s fingers to their initial state after training. For finger gesture recognition, 15 IMUs are placed on the back of the glove. Every three IMUs are connected by one flexible printed circuit (FPC) board. The finger posture angles are solved through static threshold correction and a complementary filtering algorithm. Finally, we utilize a VR rehabilitation training system with objects in different shapes for grasping, in which the patient’s hand is mapped in real-time. The VR rehabilitation training glove is a low-cost device that costs about USD150. The proposed glove system can provide patients with more attractive, personalized, and effective rehabilitation training therapy with the help of rehabilitation training theory and VR, which can enhance patients’ initiative and motivation and improve the effectiveness of rehabilitation training.

## 2. Glove System Design and Experimental Testing

### 2.1. Overview

**Figure 1:**
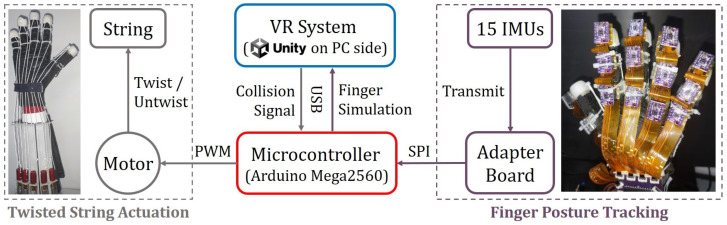
Overview of the proposed system.

Figure 1 shows an overview of the proposed VR rehabilitation training glove system. This system comprises two main subsystems: a finger posture tracking system and a twisted string actuation (TSA) system. The finger posture tracking system utilizes fifteen IMUs to monitor the real-time angles of each finger joint and is referred to as the IMU system later in the text. These angle data are transmitted to the VR system, which uses a pre-built virtual hand to simulate the physical hand based on the recorded angles. The VR system also detects collisions between the virtual hand and objects in the virtual scene. When there is no collision, the TSA system continuously drives the twisted wires, converting the motor’s rotation into linear tension on the tendons to facilitate the patient’s gripping. Conversely, when a collision is detected, the TSA system stops functioning.

The finger posture tracking subsystem includes multiple flexible printed circuits (FPCs), IMUs, and an adapter board, while the TSA actuation subsystem comprises five actuation motors, five couplings, and five pairs of force-conducting tendon wires. These two subsystems are controlled by a microcontroller unit (MCU) that communicates with a personal computer (PC) through a serial port. The IMUs are connected to the MCU via a serial peripheral interface (SPI).

When the system is activated, the IMU captures raw data that is sent to the MCU through FPC. The MCU decodes the data to obtain finger joint attitude data, which is then transmitted to a Unity VR system on the PC. The Unity VR system performs real-time simulation of the virtual hand model. Simultaneously, the TSA actuation system rotates force-conducting tendon wires and applies tension to the fingertips to aid gripping for the patient. The VR system continuously detects collisions between the virtual hand and interactive virtual objects. When a collision is detected on a particular fingertip, the Unity VR engine sends a signal to the MCU to stop the motor corresponding to that finger from working, that is, the finger stops being driven to grip.

### 2.2. Hardware Design of TSA System

**Figure 2:**
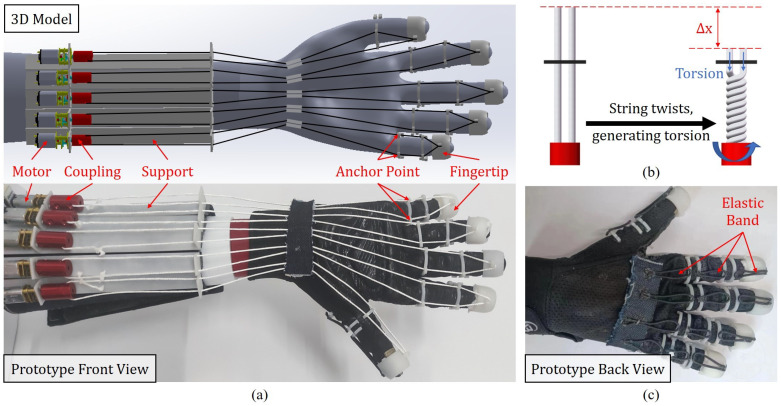
(**a**) 3D model and prototype of the TSA actuation system; (**b**) the principle of TSA; (**c**) back view of the prototype glove.

The TSA actuation system consists of five motors (CHR-GM12-N20K, CHN), five couplings, a support structure, and anchoring structures, as shown in Figure 2a. Each motor only weighs about 11 g, ensuring the lightness of the actuation system. The support structure is 3D-printed using PLA and the motors are securely positioned on the user’s arm. Moreover, a pair of small holes with fixed spacing are present on the supporting structure and above each motor, easing the passage of twisted strings. One end of the twisted strings is affixed to the 3D-printed fingertip cover, while an anchoring structure with a pair of holes serves as an anchor point at each knuckle of the glove for improved force balance. The strings are split into two parts along the fingers, pass through the holes of the anchoring structure, and ultimately connect to the coupling on the actuation end. Figure 2b illustrates the principle of TSA, where the motor, controlled by an MCU, drives the twisted string to convert rotational motion into linear tension on the twisted strings through twisting and winding. This provides assistance to the user in gripping. Figure 2c displays a back view of the glove, which incorporates elastic bands to aid the user in restoring the open position of the hand. The TSA actuation structure developed in this study is lightweight and compact, making it suitable for integration into wearable devices.

The wearable part on the arm, including the TSA system, the cuff, etc., weighs approximately 180 g in total. This weight can be further reduced using a lighter cuff. The hand-wearable part, including the glove, IMUs, accessories, etc., only weighs 110 g. The wearable part on a single hand should weigh no more than 500 g [23] to minimize obstruction of the patient’s hand movements, as satisfied by our system. Table 1 compares the weight and performance with other motor-cable-driven rehabilitation gloves. Compared to previous work, this study demonstrated a lightweight solution for rehabilitation gloves with whole-hand finger actuation and motion tracking.

### 2.3. Twisted String Actuator Testing

**Figure 3:**
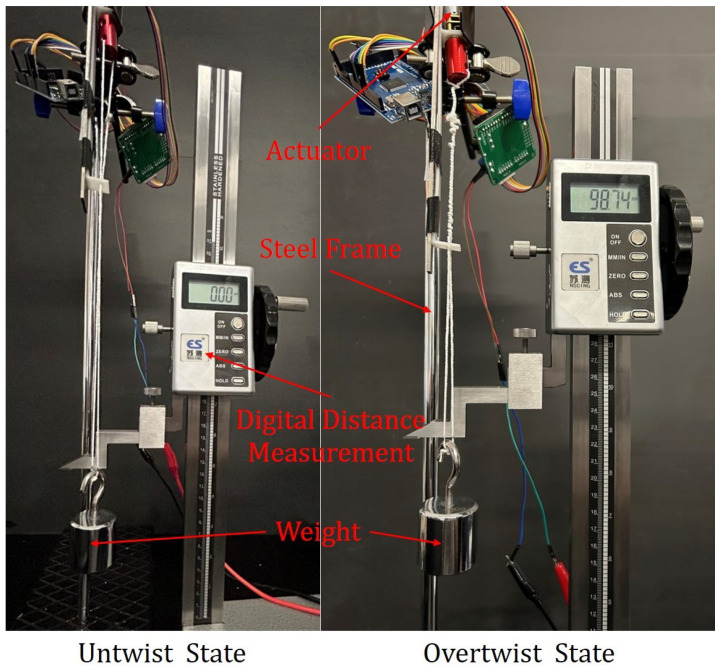
Loading test setup of the twisted string actuator.

To evaluate the performance of the TSA, we conducted experiments to measure its traveling distance, actuation force, and traveling speed. The relationship between traveling distance and time was obtained under different loading conditions with weights (100 g, 200 g, 300 g, and 400 g) at a fixed operating voltage (12 V). Figure 3 illustrates the setup, where the TSA is secured to a steel frame. The weight is fixed to the end of the strings, which are initially in a relaxed state. Upon activation, the strings rotate and contract. We recorded the increase in load over time using a digital distance measurement meter. To minimize experimental error, we performed five trials for each set of loads.

**Figure 4:**
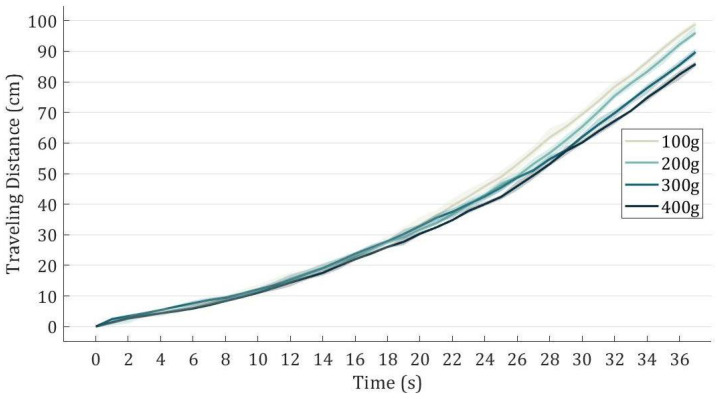
The relationship between the traveling distance and time under different loads.

Figure 4 depicts the measured correlation between the travel distance and time for various loads. Each curve represents the average values derived from five repeated tests conducted under the same load. The shaded area around each curve represents the upper and lower boundaries, indicating the range of data obtained from the five experiments conducted for each load.

The experimental results show that the curves for these loads are similar, suggesting that the actuator performs consistently within the tested range of loads, which is sufficient for actuating a single finger [27]. The TSA can achieve 80 cm in just 30 s to 36 s within the tested loading range, displaying high consistency and resulting in an average speed ranging from 2.2 cm/s to 2.7 cm/s. These results imply that the TSA actuation offers an adequate stroke, actuation force and traveling speed for hand rehabilitation purposes. The performance can be further improved by changing the motors.

### 2.4. Rehabilitation Training Gloves Testing

#### 2.4.1. Grip Force Test

**Figure 5:**
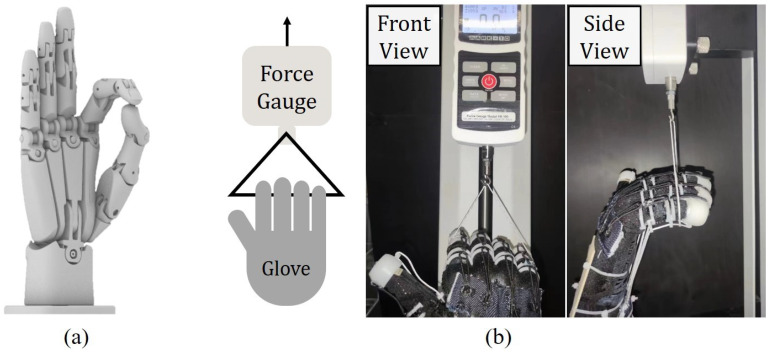
(**a**) A 3D model of the prosthetic hand; (**b**) experimental set-up of the test of the relationship between the grip force and the traveling distance.

In hand rehabilitation, the force generated by the rehabilitation equipment is essential. To evaluate the gripping force supplied by our rehabilitation gloves, we made a prosthetic hand. The prosthetic hand is 3D-printed to mimic a human hand, and has 16 degrees of freedom, as shown in Figure 5a. The original prosthetic hand design was proposed open source in [28], which was scaled down by 25% in this study to match the size of the commercial glove. All the finger joints are connected with a clearance fit, which was enabled by polishing the jointing surfaces, making the joints fully flexible with almost zero joint stiffness. The prosthetic hand fitted with the gloves was adjusted to a partially gripping position with its wrist fixed. Using a tensile testing machine (Mark-10 ESM 303, Mark-10, New York, NY, USA), the four fingers were pulled upwards by 20 mm, as shown in Figure 5b. In the meantime, the TSAs were kept stationary so the fingers remained in the same state in order to measure the gripping force.

**Figure 6:**
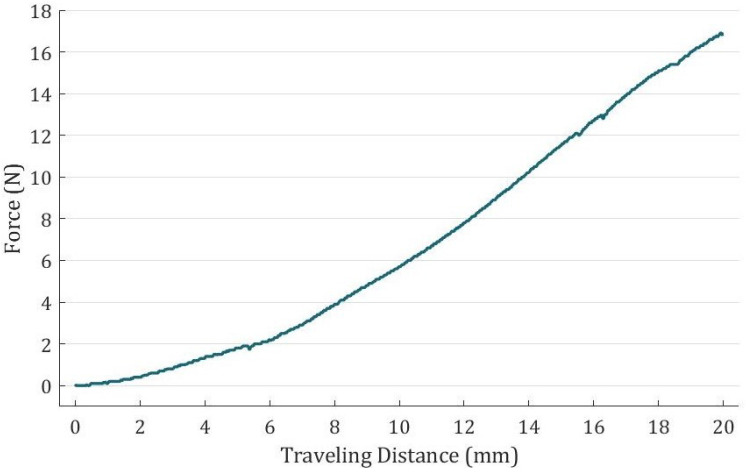
The relationship between grip force and travelling distance.

The measured relationship between the force exerted by the tensile testing machine and the traveling distance change of the glove is illustrated in Figure 6. The curve shows that the rehabilitation glove can generate a total force of 17 N across the four fingers for a traveling distance of 20 mm. This force level satisfies the grip force requirement specified by [27].

#### 2.4.2. Time Duration for Full ROM Test

**Figure 7:**
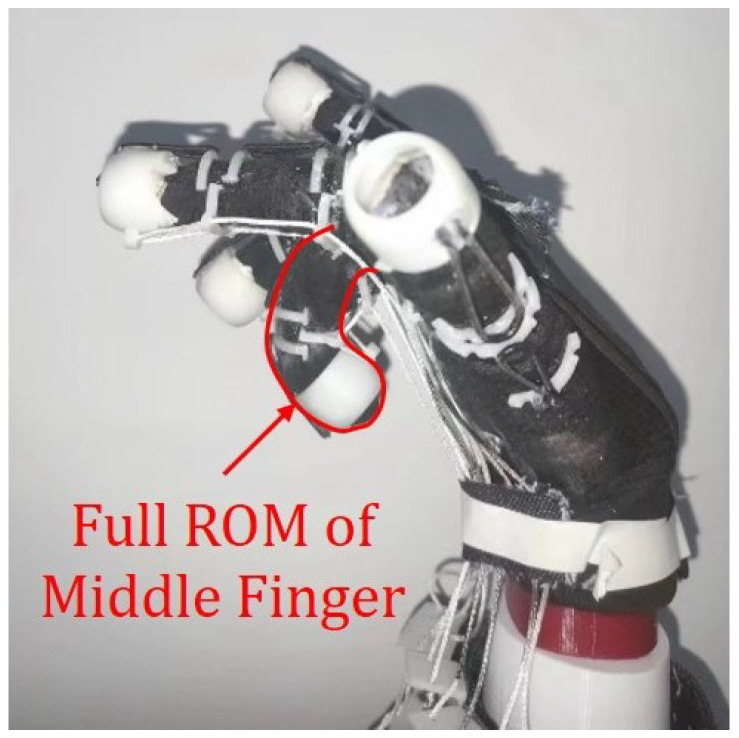
Illustration of full ROM of the middle finger.

To determine the duration of the full range of motion (ROM) for different voltages, we first adjust the fingers of the prosthetic hand with gloves to a fully grasped state (Figure 7), and mark the position on the string as the end position for subsequent experiments to ensure accuracy of the records. The experiment is divided into six groups, with voltage intervals of 0.5 V within the range of 9 V to 12 V. We recorded the time it took for the rehabilitation glove to transition from a fully open state to a fully grasping state as the finger gradually bent. Each voltage was tested five times.

**Figure 8:**
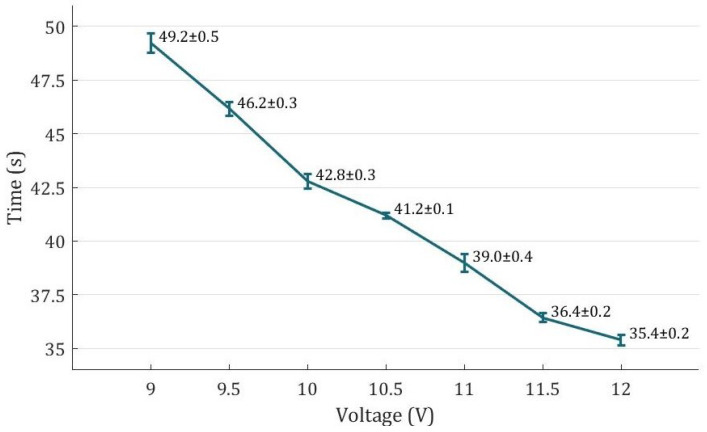
Full ROM movement time at different voltages.

Figure 8 illustrates the recorded time duration for the fingers of the rehabilitation glove to transition from fully open to fully grasping positions under various voltages. The results demonstrate that as the voltage increases, the time needed for grasping decreases, suggesting that the grasping speed can be easily controlled by adjusting the voltage. For instance, at 12 V, the glove achieves a full range of motion (ROM) in just 35.4 s. Furthermore, the standard deviations for all the voltage levels are below 0.5 s, indicating consistent performance in terms of grasping ability for the gloves.

## 3. Sensing and Control

### 3.1. Software Control Flow

**Figure 9:**
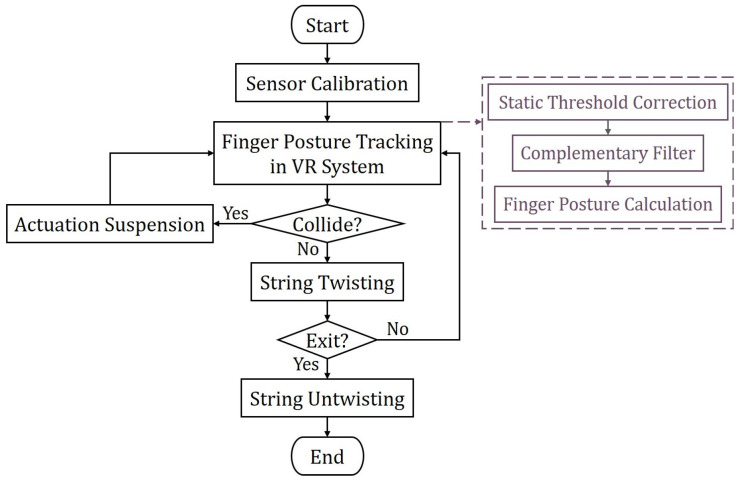
Flowchart of the proposed system.

Figure 9 illustrates the control flow of the software. Despite being of the same type, each IMU is subject to individual errors due to the manufacturing process. When the system is started, the accelerometers and gyroscopes in the six-axis IMU are first calibrated, where the IMUs are kept horizontal for a certain period of time in order to compute the average zero drift, which is later subtracted from the subsequent calculation. The MCU then performs data processing, which includes static threshold correction, complementary filter, and finger posture calculation, and demonstrates this in the real-time finger posture tracking of the VR system. During the specified grasping training task, the TSA actuation system provides assistance to the user. The VR system facilitates collision detection between the virtual hand and objects in the virtual environment. When a collision is detected, the VR system console sends a collision signal to the MCU and suspends the corresponding TSA motor. Conversely, if no collision occurs, string twisting continues to bend the finger. If all five fingers collide with the virtual object and the corresponding motors are in the suspended state, it is considered a single complete grasping training session. Eventually, when the user chooses to stop training and exit the system, the motors untwist the strings to a relaxed state.

### 3.2. IMU System and Tracking Algorithm

**Figure 10:**
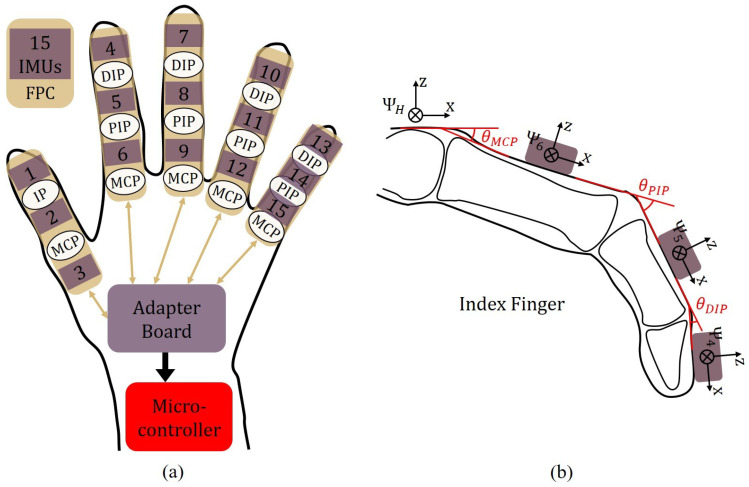
(**a**) Layout of the IMU system; (**b**) coordinate frames established to calculate the index finger posture.

In this study, the IMU system, which consists of 15 six-axis IMU (LSM6DS3, ST Microelectronics) modules, is used to measure and calculate the movement angle of each knuckle of the hand. As shown in Figure 10a, the 15 IMUs were evenly divided into five groups, and each group was attached to a finger through an FPC for connection and data transfer. The five groups of FPCs are integrated and connected to an adapter board, and then all the collected data are transmitted to the microcontroller in a unified way for subsequent data processing and posture calculation operations. In order to facilitate convenient assembly and disassembly of the IMU modules, they are connected to the circuit via a pin header design on each FPC. This design enables individual replacement of worn-out IMU modules, reduction in costs and extension of the lifespan of the glove. Additionally, the FPC is equipped in the form of a golden finger, which could be conveniently connected to the flip-type FPC connector on the adapter board, improving integration and data transfer efficiency.

Except for the thumb, the other four fingers have three phalangeal joints—from inside to outside, the metacarpal phalangeal (MCP), the proximal interphalangeal (PIP), and the distal interphalangeal (DIP). Figure 10b shows the index finger as an example, with the coordinate frames for hand $\Psi_{H}$ and the three IMUs $\Psi_{4}$, $\Psi_{5}$, $\Psi_{6}$ labeled. Beginning from the start of the system when the palm is in a horizontally fully open state, the attitude angle of the MCP $\theta_{MCP}$ is represented by the pitch angle of $\Psi_{6}$, which is the angle of rotation around its y-axis. And $\theta_{PIP}$ and $\theta_{DIP}$ are obtained by subtracting the pitch angles of two neighboring IMUs.

#### 3.2.1. Static Threshold Correction

The gyroscope in a six-axis IMU collects the real-time angular velocities ${\dot{\theta }}_{Gyro}$ in unit time step Δt during motion and then transmits the raw data to the MCU for integration to obtain the unit integration attitude angle $\theta_{Gyro}$ solved by the gyroscope alone; that is,

(1) $$\theta_{Gyro}={\dot{\theta }}_{Gyro}\cdot \Delta t$$

Compared to using a single IMU, utilizing multiple IMUs introduces the need for sequential processing and resolving data from each IMU. Consequently, this multi-IMU system requires more time and leads to the accumulation of errors. Even in scenarios where the IMUs are at rest, the integration still occurs, resulting in significant integration errors.

To address this issue, we employ the static threshold correction method proposed in our previous study [19]. During the proposed static calculation, multiple IMUs on the data glove are turned on and kept stationary simultaneously for five minutes. The $\theta_{Gyro}$ of one random IMU gyroscope is recorded during this time period. From this, we determine the range of angles obtained by this single IMU, which is then defined as the natural error interval. When the calculated angle falls within this natural error interval, it is deemed to be in a static state, and the angle is not accumulated. This approach ensures the stability of IMU calculation in a static state and mitigates the problem of error accumulation caused by angle integration. In this study, the natural error interval used is (−0.020°, 0.025°) as obtained in our previous study [19].

#### 3.2.2. Complementary Filter

Based on the coordinate frames established in Figure 10b, the attitude solution of the IMU in this study is considered to follow the moving axis of the Euler angle criterion in z-y-x order. Therefore, the required pitch angle can be calculated from the data collected by the accelerometer by the following equation:

(2) $$\theta_{Acc}=-\arctan\left(\frac{Acc_{x}}{\sqrt{Acc_{y}^{2}+Acc_{z}^{2}}}\right)$$

where $Acc_{x}$, $Acc_{y}$, and $Acc_{z}$ are the raw data collected by the accelerometer in *x*, *y*, and *z* directions.

In the study, we utilize the complementary filter to fuse the angles solved by the gyroscope and accelerometer for determining attitude. This algorithm offers several advantages, including low computational complexity, fast speed, and suitability for multi-IMU systems.

Given the non-negligible high-frequency motion perturbations of the accelerometer, the angle $\theta_{Acc}$ obtained from it is low-pass filtered. Conversely, the gyroscope’s high-frequency properties necessitate high-pass filtering of the integrated angle $\theta_{Gyro}$ to dismiss low-frequency accumulated noise. Subsequently, both angles are weighted and combined. The resulting estimated attitude angle θ is calculated as

(3) $$\theta =f\left[\theta_{prev}+\theta_{Gyro}\right]+\left(1-f\right)\theta_{Acc}$$

where $\theta_{prev}$ is the angle at the previous time step. The scaling factor, denoted as *f*, is set to 0.96 to improve estimation accuracy. Once the estimated attitude angle of each IMU is obtained, the angle of each finger joint can be calculated from the difference between two neighboring IMU angles, as described in Figure 10b.

### 3.3. Real-Time VR Application

**Figure 11:**
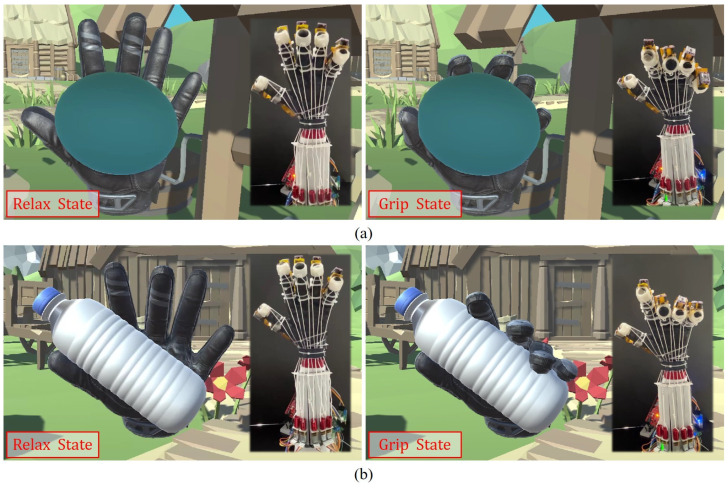
The proposed VR rehabilitation training system: (**a**) gripping a ball; (**b**) gripping a bottle.

A VR rehabilitation training system was developed based on the control flow depicted in Figure 9. In this system, we perform real-time VR application experiments using the prototype rehabilitation training glove. Figure 11 demonstrates the user’s hand interacting with a ball and a bottle in the Unity-based VR system. The continuous and detailed process can be seen in Supplementary Video S1.

Hand gesture data collected from multiple IMUs is processed by the MCU and then sent to the VR system on the PC through a USB serial port. The VR system uses this data to simulate the movement of corresponding joints in the virtual hand, enabling real-time gesture recognition and mapping.

In addition, a certain volume of the touch zone is assigned to both the interactive object and the fingertips of the virtual hand, respectively. We determine that the virtual hand and the interactive object collide when the touching zones of the two intersect. When the software is running, the VR system continuously checks for collisions between the virtual hand and the objects that can be interacted with. In the absence of any collisions, the TSA actuation system operates continuously. This system causes the strings to twist, creating tension in the user’s fingers for grasp training. When a collision between the virtual hand and an interactive object is detected, the VR system sends a signal to the MCU via a serial port. The motor controlling the finger that collided with the object stops working and the finger of the virtual hand remains on the surface of the object, resulting in a successful virtual grasp.

## 4. Conclusions and Future Work

This study proposes a VR rehabilitation training glove with both twisted string actuation (TSA) actuation and finger posture recognition using inertial measurement units (IMUs). The glove, including the entire actuation system, is lightweight (less than 300 g) and affordable (about USD150). As a cost-effective and portable VR rehabilitation device, it has great potential to enhance patients’ rehabilitation outcomes through diverse games and interactive activities.

This paper provides detailed information on the principles and algorithms of TSA actuation assistance for grip training and finger posture recognition for motion tracking. A series of experiments were conducted to demonstrate the feasibility of the rehabilitation training glove. The glove achieved a full range of motion (ROM) within 35 s, exerting a total force of 17 N force on all fingers, except the thumb, with a traveling distance of 20 mm. These results show that the TSA actuation method can provide appropriate stroke, force, and speed for effective rehabilitation. Real-time VR application experiments also confirm the feasibility and effectiveness of the rehabilitation glove in the VR environment.

Nonetheless, this research can still be improved in several aspects. The current study utilizes the elastic bands integrated at the back of the fingers to generate torsional stiffness to prevent the TSAs from damaging the fingers. In the future, we plan to optimize the torsional stiffness [29] of the glove to further improve safety and comfort. In addition, the speed of the TSAs will be increased by choosing motors with a lower reduction ratio. Other strategies will include integrating artificial intelligence algorithms to enhance the system’s intelligence and personalization capabilities. Clinical trials and further improvements will also be conducted to validate the VR rehabilitation training glove’s effectiveness.

## Figures and Tables

**Table 1:** Comparison of motor-cable-driven rehabilitation gloves.

References	Year	Hand (g)	Arm (g)	Total Weight on Upper Limb (g)	Driven Method	Actuation Degree of Freedom ^1^	Finger Motion Tracking
Mohammadi et al. [24]	2018	-	-	330	Cable+spool	4	Motion classification (sEMG sensor)
Cheng et al. [25]	2018	206	-	206	Cable+spool	5	Index finger (curvature sensor)
Zhou et al. [26]	2019	-	-	500	Cable+spool	2	Thumb and index fingers (IMU)
Tsabedze et al. [13]	2021	186	-	-	TSA	4	Four larger fingers (SCP strings displacement sensing)
This study	2023	110	180	290	TSA	5	All fingers (IMU)

^1^ “Actuation Degree of Freedom” refers to how many fingers the system can independently actuate.

## Data Availability

The data that support the findings of this study are available from the authors upon reasonable request.

## Supplementary Materials

The following supporting information can be downloaded at: https://www.mdpi.com/article/10.3390/biomimetics8050425/s1, Video S1: Demonstration of real-time VR rehabilitation training.
